# The association between phthalates and metabolic syndrome: the National Health and Nutrition Examination Survey 2001–2010

**DOI:** 10.1186/s12940-016-0136-x

**Published:** 2016-04-14

**Authors:** Tamarra M. James-Todd, Tianyi Huang, Ellen W. Seely, Aditi R. Saxena

**Affiliations:** Departments of Environmental Health and Epidemiology, Harvard T.H. Chan School of Public Health, 665 Huntington Ave., Bldg. 1, 14th Floor, Boston, MA 02115 USA; Division of Women’s Health, Department of Medicine, Connors Center for Women’s Health and Gender Biology, 1620 Tremont St., 3rd Floor, Boston, MA 02115 USA; Channing Division of Network Medicine, Brigham and Women’s Hospital and Harvard Medical School, 181 Longwood Ave., Boston, MA 02115 USA; Division of Endocrine, Diabetes, and Hypertension, Brigham and Women’s Hospital and Harvard Medical School, 221 Longwood Ave., 3rd Floor, Boston, MA 02115 USA

**Keywords:** Phthalates, Metabolic syndrome, Blood pressure, Obesity, Cholesterol, Sex

## Abstract

**Background:**

Higher exposure to certain phthalates is associated with a diabetes and insulin resistance, with sex differences seen. Yet, little is known about the association between phthalates and metabolic syndrome (MetS), particularly with consideration for differences by sex and menopausal status.

**Methods:**

We analyzed data from 2719 participants in the National Health and Nutrition Examination Survey (NHANES) 2001–2010 aged 20–80 years. Five urinary phthalate metabolites (MEP, MnBP, MiBP, MBzP, and MCPP) and DEHP metabolites were analyzed by the Centers for Disease Control and Prevention and were evaluated as population-specific quartiles. MetS was defined by National Cholesterol Education Program’s Adult Treatment Panel III report criteria. Prevalence odds ratios (POR) and 95 % confidence intervals (CI) were calculated using multivariable logistic regression, adjusting for potential confounders and stratifying by sex and menopausal status.

**Results:**

Participants with MetS (32 % of the study population) had higher concentrations for all urinary phthalate metabolites. After full adjustment, higher DEHP metabolite concentrations were associated with an increased odds of MetS in men, but not women (adj. POR for men Q4 versus Q1: 2.20; 95 % CI: 1.32, 3.68 and adj. POR for women Q4 versus Q1: 1.50; 95 % CI: 0.89, 2.52). When evaluating by menopausal status, pre-menopausal women with higher concentrations of MBzP had close to a 4-fold increased odds of MetS compared to pre-menopausal women with the lowest concentrations of MBzP (adj POR: Q4 versus Q1: 3.88; 95 % CI: 1.59, 9.49).

**Conclusions:**

Higher concentrations of certain phthalate metabolites were associated with an increased odds of MetS. Higher DEHP metabolite concentrations were associated with an increased odds of MetS for men. In women, the strongest association was between higher concentrations of MBzP and MetS, but only among pre-menopausal women.

**Electronic supplementary material:**

The online version of this article (doi:10.1186/s12940-016-0136-x) contains supplementary material, which is available to authorized users.

## Background

There is a growing body of predominantly cross-sectional epidemiologic studies examining the association of phthalate exposure with obesity [1–3], insulin resistance [4–6], and blood pressure [7–9]. Little is known about the association of phthalate exposure and metabolic syndrome (MetS). Comprised of central obesity, hypertriglyceridemia, hyperlipidemia, insulin resistance, and hypertension [10, 11], MetS represents an integrated measure of metabolic dysfunction. Captured by this constellation of commonly measured clinical variables, MetS is associated with an increased risk for cardiovascular disease and all-cause mortality by 50–200 % [12–15]. Lifestyle factors [16] and socioeconomic status [17, 18] are strong predictors of MetS, and the prevalence of the condition varies by sex and race/ethnicity [19, 20]. Based on the National Health and Nutrition Examination Survey (NHANES), a nationally-representative sample from the U.S. population between 2003 and 2013, the prevalence of MetS is 33 % [21]. While obesity and insulin resistance are components of MetS and are independently associated with increased cardiovascular disease risk, MetS is a better predictor of cardiovascular disease than its individual components alone [22, 23]. As such evaluating the role of phthalates on MetS may provide insight into whether higher exposure to these chemicals might be associated with MetS, and thereby increasing cardiovascular disease risk.

As a class of endocrine disrupting chemicals, phthalates are found in a variety of consumer products, including food packaging, cosmetics, and vinyl flooring [24]. Phthalate levels vary across the population, with women and racial/ethnic minorities having higher concentrations of these chemicals compared to men and whites [25, 26]. These differences in urinary phthalate metabolite concentrations across the population may be attributed to differences in behavioral patterns and lifestyle factors that can alter phthalate exposure, such as personal care product [27] and medication use [28], as well as processed foods packaged in high molecular weight phthalate-containing plastic [24]. For example, it is hypothesized that women have higher concentrations of certain urinary phthalate metabolites compared to men due to their use of perfumes, nail polishes, and other personal care products. A previous study showed significant sex-phthalate interactions for ∑DEHP and fasting blood glucose, as well as MBzP and fasting insulin. Patterns of association were different, with stronger associations seen in men for ∑DEHP and fasting glucose compared to women. The opposite was true for the association between MBzP and fasting insulin in women compared to associations seen in men [4, 6]. Little is known about the association between phthalates and other components of MetS, namely hypertension, low HDL and hypertriglyceridemia or with MetS as a whole.

Phthalates are thought to contribute to obesity and increased insulin resistance through a number of pathways. For example, phthalates are known to bind to peroxisome proliferator-activated receptor gamma (PPAR-γ), which could upregulate adipocyte production and, in an environment with excess caloric intake, could contribute to insulin resistance [29]. Phthalates are also known to modulate hormones and inflammatory pathways [30], which could subsequently lead to increased inflammatory profile and insulin resistance [31]. Both adipogenic and inflammatory pathways are known to be involved in MetS [32]. As such, higher phthalate exposure could affect adipogenic or inflammatory pathways to alter risk of MetS.

Given that the association between phthalates and MetS remains unclear, we conducted an exploratory analysis using data from the National Health and Nutrition Examination Survey (NHANES) to evaluate the association between eight phthalate metabolites and MetS. We posited that associations between phthalate metabolite concentrations and MetS would differ by sex based on reported differences in the association of phthalate metabolite concentrations with insulin resistance by sex [4]. Given that menopause marks a time period of increased incidence of MetS [33, 34], we also hypothesized that the association between phthalate metabolites and MetS might differ based on menopause status.

## Methods

### Study population

The National Health and Nutrition Examination Survey (NHANES) is an ongoing survey conducted by the National Center for Health Statistics. NHANES is a nationally representative sample of the U.S. non-institutionalized civilian population. A complex, multistage probability sampling strategy is used, which oversamples certain subgroups of the population, including blacks, Mexican-Americans, and those of lower socioeconomic status. Each year approximately 5000 individuals participate in NHANES, and the data are reported in 2-year cycles available for public use. Demographic, dietary, and behavioral information is collected through in-home questionnaires, while trained assistants collect anthropometric and biomarker data using mobile exam units.

We identified 3431 participants aged 20–80 with single measurements of urinary phthalate metabolites and data on all five components for MetS. We excluded 396 with missing covariate information, 131 pregnant or lactating women and 185 participants with fasting time <8 hours. These exclusions resulted in an analytic dataset of 2719 adults. Data were pooled from NHANES 2001–2010, with analysis restricted to 2719 men and non-pregnant and non-lactating women aged 20–80 years who had fasted for ≥8 hours and had complete information on phthalate exposure, outcome and covariates. As a part of the NHANES protocol, informed consent was obtained in writing from all study participants. This study was approved by Brigham and Women’s Hospital’s Partners Human Research Committee.

### Measurement of phthalate metabolites

In a random one-third subsample of NHANES study participants, phthalate metabolites were measured in a spot urine sample. These samples are collected and frozen at -20C and shipped to the National Center for Environmental Health at the Centers for Disease Control for analysis of a variety of phthalate metabolites. Phthalate metabolites are measured instead of their parent compounds to lower the potential for exposure misclassification. A combination of solid phase extraction, high pressure liquid chromatography, and tandem mass spectrometry was used to measure phthalate metabolite levels using methods described elsewhere [26].

The phthalate metabolites measured in NHANES study participants varied by year. We selected a combination of those that had been measured in previous studies showing associations with metabolic derangements, as well as those that were measured in all years with >60 % of sample concentrations above the limit of detection (LOD) [35]. Based on these criteria, we evaluated mono-ethyl phthalate (MEP), mono-n-butyl phthalate (MnBP), mono-isobutyl phthalate (MiBP), mono-benzyl phthalate (MBzP), mono-(3-carboxypropyl) phthalate (MCPP), and di(2-ethylhexyl) phthalate (DEHP) metabolites (i.e. mono-(2-ethylhexyl) phthalate (MEHP), mono-(2-ethyl-5-hydroxyhexyl) phthalate (MEHHP) and mono-(2-ethyl-5-oxohexyl) phthalate (MEOHP)). Given that the DEHP metabolites were highly correlated, we used the molar sum of these metabolites, denoted by ΣDEHP. To allow potential non-linear associations as previously found, we evaluated individual phthalate metabolites and ΣDEHP using population-specific quartiles. The lowest quartile was considered the referent category. Those measured concentrations below LOD were replaced with LOD divided by square root of two, based on previous analyses [35].

### Metabolic syndrome

We used the National Cholesterol Education Program’s Adult Treatment Panel III report (NCEP/ATPIII) to define MetS [36]. The NCEP/ATPIII classifies an individual as having MetS, if at least 3 of the following 5 criteria are satisfied: 1) waist circumference ≥ 102 cm in men or ≥ 88 cm in women; 2) triglycerides ≥ 150 mg/dL; 3) high density lipid (HDL) cholesterol < 40 mg/dL in men or <50 mg/dL in women; 4) blood pressure ≥ 130/85 mmHg or treatment for hypertension; 5) fasting blood glucose ≥ 100 mg/dL or treatment for diabetes. Data on waist circumference and blood pressure were collected from NHANES and details are provided in the NHANES Anthropometry Procedures Manual [37]. The average of all available measures after a total of 4 attempted readings was used to indicate participants’ blood pressure levels. Fasting blood glucose, HDL cholesterol, and triglyceride levels were assayed using methods described in the NHANES Laboratory/Medical Technologists Procedures Manual [38]. Information on antihypertensive or anti-diabetic medication was self-reported and collected from study questionnaires.

### Covariates

Age, sex, race/ethnicity, physical activity, smoking status, educational attainment, poverty status, total caloric intake, and menopausal status in women were self-reported by questionnaire. Race/ethnicity was classified as white (referent), black, Mexican-American, or others. Age was evaluated by 10-year age groups including 20- < 30, 30- < 40, 40- < 50 (referent), 50- < 60, 60- < 70, or 70- < 80 years. The upper age limit was restricted to <80 years due to NHANES classifying participants who were age >80 years as 80 or 85 in some surveys to protect confidentiality. Physical activity was assessed as self-reported participation in vigorous, moderate or none (referent) recreational physical exercise. Smoking status was categorized as current, past, or never (referent) smoker and educational attainment as high school graduate or less (reference), some college, or college graduate or higher. Poverty status was defined by income-to-poverty ratio <1 (below the poverty level) versus ≥1 (reference) based on the federal poverty threshold and household income information [39]. Women were considered as postmenopausal if they reported no regular menstrual periods due to menopause or surgery. Further, total caloric intake was derived from a one-time 24 h dietary recall in 2001–2002 and from the average of two-day 24 h recalls in 2003–2010. Total caloric intake was divided into quartiles, with the lowest quartile considered as the referent category.

### Statistical analysis

For all statistical analyses, sampling weights were included in the dataset and applied in procedures available in SAS 9.3 (Cary, NC) that appropriately accounted for the stratification and complex sampling of NHANES. These procedures included PROC SURVEYMEANS, PROC SURVEYFREQ and PROC SURVEYLOGISTIC. First, weighted means/standard errors and numbers/percentages were calculated to describe demographic, behavioral and metabolic differences between individuals with and without MetS. Levels of log-transformed concentrations of urinary phthalate metabolites were evaluated by MetS status using weighted geometric means and 95 % confidence intervals (CIs).

Next, logistic regression was used to estimate prevalence odds ratios (PORs) and 95 % CIs as a cross-sectional measure of associations between phthalate metabolites and MetS. Model 1 adjusted for urinary creatinine, as duration of urine collection may influence measured urinary concentrations of phthalate metabolites. Model 2 adjusted for potential confounders, including age, sex, race/ethnicity, total caloric intake, education, physical activity, smoking, and poverty level. For these analyses, we used traditional methods to adjust for creatinine [1, 4–6]. We also evaluated associations between each urinary phthalate metabolite and each component of MetS using the above-specified models. To determine whether there was an association between urinary phthalate metabolite concentrations and the number of MetS components we used multivariable ordinal logistic regression, adjusting for age, sex, race/ethnicity, total caloric intake, education, physical activity, smoking, and poverty level. Given that the pathophysiology of MetS may differ by sex, similar statistical modeling was performed separately in men and women.

Both age and menopausal status are important predictors of MetS [33, 34]. In order to evaluate whether the association between phthalates and MetS differed by age, we evaluated this association stratified by <50 years vs. ≥50 years for men and women separately. To explore potential differences by menopausal status, we evaluated the association between phthalates and MetS in pre- versus post-menopausal women.

## Results

Of the 2719 adults in the study population, a total of 31.8 % of the participants (31.7 % in men and 31.9 % in women) met NCEP/ATPIII criteria for MetS. Individuals with MetS were older, more likely to be physically inactive (*p* < 0.0001), less educated (*p* < 0.0001), and ever smokers (*p* = 0.04) (Table 1). For men < 50 years old, the prevalence of MetS was 23.6 %, compared with 44.0 % in men ≥ 50 years old. For women <50 years old, the prevalence of MetS was 21.1 %, while 47.4 % of women ≥ 50 years had MetS. For premenopausal women in this study population, the prevalence of MetS was 21.9 %, compared with 46.8 % in postmenopausal women.

**Table 1:** Population characteristics by metabolic syndrome status in men and women

	Men (*n* = 1388)		Women (*n* = 1331)	
	MetS	No MetS	MetS	No MetS
	(*n* = 464)	(*n* = 924)	(*n* = 501)	(*n* = 830)
	Mean (SE)			
Age (yrs)*, ***	51.7 (0.7)	42.7 (0.6)	52.6 (0.8)	42.8 (0.5)
BMI (kg/m^2^)*, ***	32.8 (0.3)	26.7 (0.2)	33.3 (0.4)	26.6 (0.2)
Waist circumference (cm)*, ***	114.0 (0.7)	95.8 (0.5)	107.2 (0.7)	89.3 (0.6)
Diastolic blood pressure (mmHg)	74 (0.7)	70 (0.5)	71 (0.7)	69 (0.4)
Systolic blood pressure (mmHg)*, ***	129 (0.9)	119 (0.6)	126 (0.9)	115 (0.7)
Triglycerides*, ***	250 (19.3)	124 (3.0)	188 (7.1)	98 (2.3)
HDL*, ***	41 (0.8)	51 (0.5)	49 (0.8)	63 (0.6)
Glucose*, ***	121 (2.0)	100 (0.7)	115 (1.8)	92 (0.4)
Urinary creatinine (mg/dL)	150 (4.0)	153 (3.3)	119 (2.9)	110 (3.5)
Total caloric intake (kcal)****	2573 (57)	2616 (38)	1691 (35)	1820 (30)
	N (%^a^)			
Age*, ***				
< 50 year	162 (44.7)	566 (67.3)	177 (39.1)	549 (68.3)
≥ 50 year	302 (55.3)	358 (32.7)	324 (60.9)	281 (31.7)
Menopausal status, ***				
Premenopausal	--	--	181 (41.1)	544 (68.7)
Postmenopausal	--	--	320 (58.9)	286 (31.3)
Race/ethnicity*				
Mexican-American	103 (8.2)	189 (9.8)	117 (8.3)	138 (6.0)
White	278 (81.5)	447 (69.8)	229 (69.3)	431 (72.6)
Black	56 (5.1)	191 (10.7)	103 (12.2)	154 (10.5)
Other	27 (5.2)	97 (9.7)	52 (10.2)	107 (10.9)
Physical activity*, ***				
Vigorous	111 (25.0)	370 (43.5)	74 (15.7)	216 (29.1)
Moderate	138 (33.7)	194 (20.8)	147 (34.3)	237 (28.9)
No	215 (41.2)	360 (35.6)	280 (49.9)	377 (42.0)
Smoking**				
Never	189 (45.3)	426 (46.1)	277 (52.3)	499 (56.4)
Current	102 (21.8)	261 (28.8)	106 (23.2)	167 (22.2)
Past	173 (32.9)	237 (25.2)	118 (24.5)	164 (21.4)
Education**, ***				
High school or less	266 (48.4)	462 (40.9)	313 (52.3)	347 (33.7)
Some college	106 (27.5)	244 (28.5)	126 (29.3)	274 (34.8)
College graduates or higher	92 (24.1)	218 (30.6)	62 (18.4)	209 (31.5)
Poverty status****				
Yes	64 (8.8)	147 (10.6)	139 (20.6)	146 (12.3)
No	400 (91.2)	777 (89.4)	362 (79.4)	684 (87.7)

*p < 0.0001 in men; **p < 0.05 in men; ***p < 0.0001 in women; ****p < 0.05 in women

^a^Weighted percentages after accounting for the sampling design

While materially urinary phthalate metabolite concentrations appeared somewhat higher in the MetS group compared to the group without MetS, we found that only concentrations of MCPP reached borderline significance higher in the MetS men and women compared to those without MetS (*p* = 0.05) (Table 2). In the overall study population, there was a modest positive association between MBzP and MetS. After full adjustment there was a ~40 % increased odds of MetS among those with higher MBzP levels compared to those with the lowest levels (Q1) (adj. POR for Q2: 1.40 (95 % CI: 1.04, 1.89); adj. POR for Q3: 1.37 (95 % CI: 0.96, 1.96); and adj. POR for Q4: 1.44 (95 % CI: 0.98, 2.12)) (Table 3). This association was largely due to the positive associations observed in women (adj. POR for Q2: 1.55 (95 % CI: 1.07, 2.26); adj. POR for Q3: 1.71 (95 % CI: 1.05, 2.80); and adj. POR for Q4: 1.69 (95 % CI: 0.97, 2.93) compared with Q1. In contrast, no clear association was observed between MBzP and MetS in men. On the other hand, there was a significant positive association between ∑DEHP exposure and MetS for each of the higher quartiles relative to the lowest quartile, with Q2 and Q3 being associated with a 64 % (adj. 95 % CI for Q2: 1.24, 2.17 and adj. 95 % CI for Q3: 1.22, 2.22) increased odds of MetS. Those with the highest concentrations of ∑DEHP had a 94 % increased odds of MetS (adj. 95 % CI for Q4: 1.35, 2.80). These associations were stronger in men than for women. We did not observe significant associations for MetS with other phthalate metabolites. Also, given that DEHP and MBzP are moderately correlated, when simultaneously adjusted for in our models, similar associations between DEHP and MetS were found, but associations between MBzP and MetS were attenuated and became non-significant.

**Table 2:** Urinary phthalate metabolites by metabolic syndrome status

	Metabolic syndrome	No metabolic syndrome	*p*-value
	(*n* = 965)	(*n* = 1754)	
	Geometric mean (95 % CI)		
MEP	161.8 (140.7, 186.1)	142.9 (129.5, 157.7)	0.10
MBzP	10.4 (9.4, 11.7)	9.6 (8.7, 10.5)	0.26
MnBP	17.2 (15.7, 18.9)	16.2 (15.1, 17.5)	0.33
MCPP	2.8 (2.5, 3.1)	2.5 (2.3, 2.6)	0.05
MiBP	4.7 (4.2, 5.3)	4.4 (4.1, 4.7)	0.37
∑DEHP	0.13 (0.12, 0.15)	0.12 (0.10, 0.13)	0.06

**Table 3:** Prevalence odds ratios and 95 % confidence intervals for metabolic syndromes according to urinary phthalate metabolites in men and women

	Overall population		Men		Women	
	(*n* = 2719)		(*n* = 1388)		(*n* = 1331)	
	Model 1^a^	Model 2^b^	Model 1^a^	Model 2^c^	Model 1^a^	Model 2^c^
MEP						
Q1	Ref	Ref	Ref	Ref	Ref	Ref
Q2	1.07 (0.82, 1.40)	1.16 (0.88, 1.54)	0.92 (0.63, 1.34)	0.98 (0.66, 1.45)	1.21 (0.84, 1.75)	1.46 (0.97, 2.19)
Q3	0.94 (0.69, 1.27)	0.98 (0.71, 1.37)	0.97 (0.62, 1.53)	0.97 (0.58, 1.61)	0.86 (0.59, 1.27)	1.02 (0.66, 1.56)
Q4	1.20 (0.91, 1.59)	1.18 (0.88, 1.59)	1.33 (0.90, 1.96)	1.35 (0.87, 2.11)	1.04 (0.71, 1.51)	1.11 (0.72, 1.72)
MBzP						
Q1	Ref	Ref	Ref	Ref	Ref	Ref
Q2	1.33 (1.00, 1.75)	1.40 (1.04, 1.89)	1.25 (0.83, 1.87)	1.19 (0.75, 1.87)	1.34 (0.96, 1.88)	1.55 (1.07, 2.26)
Q3	1.27 (0.92, 1.78)	1.37 (0.96, 1.96)	1.04 (0.68, 1.59)	1.01 (0.63, 1.63)	1.49 (0.95, 2.35)	1.71 (1.05, 2.80)
Q4	1.27 (0.86, 1.86)	1.44 (0.98, 2.12)	1.03 (0.62, 1.70)	1.08 (0.65, 1.79)	1.46 (0.90, 2.36)	1.69 (0.97, 2.93)
MnBP						
Q1	Ref	Ref	Ref	Ref	Ref	Ref
Q2	1.23 (0.92, 1.66)	1.21 (0.90, 1.63)	1.10 (0.73, 1.64)	1.06 (0.71, 1.58)	1.35 (0.95, 1.91)	1.31 (0.90, 1.89)
Q3	0.97 (0.71, 1.33)	0.94 (0.67, 1.33)	0.66 (0.41, 1.07)	0.57 (0.34, 0.96)	1.37 (0.99, 1.90)	1.45 (0.98, 2.16)
Q4	1.23 (0.88, 1.73)	1.22 (0.85, 1.75)	1.37 (0.87, 2.17)	1.43 (0.85, 2.40)	1.09 (0.69, 1.74)	1.03 (0.60, 1.77)
MCPP						
Q1	Ref	Ref	Ref	Ref	Ref	Ref
Q2	1.09 (0.82, 1.45)	1.05 (0.77, 1.44)	1.09 (0.74, 1.61)	0.96 (0.62, 1.49)	1.04 (0.71, 1.52)	1.05 (0.71, 1.57)
Q3	1.02 (0.72, 1.43)	0.94 (0.65, 1.34)	0.91 (0.60, 1.38)	0.75 (0.48, 1.18)	1.07 (0.68, 1.68)	0.97 (0.57, 1.66)
Q4	1.25 (0.91, 1.72)	1.12 (0.80, 1.56)	1.30 (0.85, 2.01)	0.99 (0.63, 1.58)	1.11 (0.72, 1.70)	1.08 (0.68, 1.71)
MiBP						
Q1	Ref	Ref	Ref	Ref	Ref	Ref
Q2	1.04 (0.78, 1.38)	1.07 (0.78, 1.47)	0.91 (0.64, 1.30)	0.94 (0.63, 1.38)	1.14 (0.73, 1.78)	1.13 (0.71, 1.81)
Q3	0.88 (0.64, 1.20)	0.88 (0.61, 1.27)	0.72 (0.46, 1.12)	0.72 (0.43, 1.19)	1.03 (0.66, 1.61)	1.04 (0.64, 1.70)
Q4	1.14 (0.86, 1.52)	1.28 (0.91, 1.80)	0.98 (0.69, 1.40)	1.05 (0.66, 1.65)	1.25 (0.74, 2.09)	1.40 (0.77, 2.55)
∑DEHP						
Q1	Ref	Ref	Ref	Ref	Ref	Ref
Q2	1.62 (1.25, 2.11)	1.64 (1.24, 2.17)	1.69 (1.23, 2.34)	1.60 (1.11, 2.31)	1.52 (1.07, 2.17)	1.58 (1.09, 2.29)
Q3	1.55 (1.16, 2.09)	1.64 (1.22, 2.22)	1.71 (1.17, 2.51)	1.69 (1.16, 2.46)	1.38 (0.86, 2.22)	1.43 (0.89, 2.29)
Q4	1.59 (1.12, 2.25)	1.94 (1.35, 2.80)	1.87 (1.19, 2.94)	2.20 (1.32, 3.68)	1.27 (0.78, 2.06)	1.50 (0.89, 2.52)

^a^Adjusted for urinary creatinine

^b^Adjusted for urinary creatinine, age, sex, race/ethnicity, total caloric intake, education, physical activity, smoking, and poverty

^c^Adjusted for the same variables in b except sex

Overall the weighted proportions of study subjects who met criteria for each individual component of MetS were: 52.5 % for waist circumference; 30.0 % for triglycerides; 28.9 % for HDL cholesterol; 28.6 % for blood pressure; and 42.5 % for fasting blood glucose. Only the associations between MBzP and ∑DEHP and individual components of MetS are presented, as only these phthalate metabolites were associated with MetS (Table 4; See the Additional file 1: Table S1 for the association between other urinary phthalate metabolites and MetS components). In the overall population, we found the highest quartile of MBzP had a significant association with central obesity (defined as waist circumference ≥ 102 cm men or ≥ 88 cm in women) and hypertriglyceridemia, whereas higher ΣDEHP was associated with central adiposity, hypertriglyceridemia, high blood pressure, and hyperglycemia (Table 4). When evaluating the association between urinary phthalate metabolite concentrations and the number of metabolic components, we found that the highest quartiles of MBzP, MCPP, MiBP were associated with an increased odds of having more metabolic abnormalities. Higher ∑DEHP concentrations (those with concentrations in Q2, Q3, and Q4) were associated with an increased odds of having more metabolic abnormalities (Additional file 1: Table S2).

**Table 4:** Prevalence odds ratios and 95 % confidence intervals for individual components of metabolic syndrome according to urinary phthalate metabolite levels

	Individual metabolic abnormalities of metabolic syndrome				
	Central obesity	Hypertriglyceridemia	Low HDL cholesterol	High blood pressure	Hyperglycemia
	Overall population				
# with condition /total	1522/2719	849/2719	816/2719	871/2719	1289/2719
MBzP^a^					
Q1	Ref	Ref	Ref	Ref	Ref
Q2	1.23 (0.93, 1.62)	1.20 (0.95, 1.51)	1.13 (0.81, 1.57)	0.87 (0.62, 1.21)	1.03 (0.75, 1.41)
Q3	1.34 (0.98, 1.85)	1.12 (0.84, 1.49)	1.12 (0.77, 1.63)	1.07 (0.68, 1.67)	1.36 (0.98, 1.88)
Q4	1.84 (1.31, 2.58)	1.50 (1.10, 2.05)	1.27 (0.86, 1.89)	1.18 (0.79, 1.75)	1.20 (0.80, 1.79)
∑DEHP^a^					
Q1	Ref	Ref	Ref	Ref	Ref
Q2	1.40 (1.02, 1.92)	1.34 (1.01, 1.79)	1.35 (1.01, 1.80)	1.34 (0.96, 1.88)	1.20 (0.88, 1.64)
Q3	1.31 (0.95, 1.81)	1.33 (0.96, 1.82)	0.99 (0.74, 1.31)	1.28 (0.90, 1.81)	1.60 (1.13, 2.26)
Q4	1.66 (1.16, 2.36)	1.55 (1.12, 2.14)	1.05 (0.74, 1.49)	1.56 (1.14, 2.12)	1.66 (1.13, 2.44)
	Men				
# with condition/total men	639/1388	465/1388	357/1388	437/1388	758/1388
MBzP^b^					
Q1	Ref	Ref	Ref	Ref	Ref
Q2	1.20 (0.75, 1.90)	1.21 (0.89, 1.66)	1.35 (0.88, 2.08)	0.84 (0.56, 1.27)	0.68 (0.40, 1.18)
Q3	1.11 (0.67, 1.83)	1.09 (0.75, 1.57)	1.14 (0.64, 2.04)	0.78 (0.47, 1.31)	0.96 (0.54, 1.38)
Q4	1.71 (1.06, 2.77)	1.28 (0.84, 1.95)	1.02 (0.62, 1.70)	0.89 (0.54, 1.48)	0.86 (0.48, 1.53)
∑DEHP^b^					
Q1	Ref	Ref	Ref	Ref	Ref
Q2	1.42 (0.97, 2.07)	1.13 (0.75, 1.71)	1.17 (0.69, 1.98)	1.59 (1.05, 2.40)	1.15 (0.76, 1.74)
Q3	1.24 (0.79, 1.93)	1.34 (0.89, 2.02)	1.02 (0.62, 1.68)	1.38 (0.88, 2.16)	1.57 (0.95, 2.58)
Q4	1.68 (1.02, 2.79)	1.55 (0.97, 2.49)	0.98 (0.53, 1.80)	1.85 (1.12, 3.05)	1.67 (0.96, 2.93)
	Women				
# with condition /total women	883/1331	384/1331	459/1331	434/1331	531/1331
MBzP^b^					
Q1	Ref	Ref	Ref	Ref	Ref
Q2	1.16 (0.79, 1.69)	1.17 (0.79, 1.73)	0.93 (0.60, 1.42)	0.82 (0.50, 1.36)	1.52 (1.05, 2.18)
Q3	1.46 (0.96, 2.22)	1.16 (0.71, 1.87)	1.06 (0.67, 1.67)	1.37 (0.74, 2.56)	2.06 (1.31, 3.22)
Q4	1.71 (1.07, 2.73)	1.75 (0.98, 3.13)	1.47 (0.84, 2.56)	1.40 (0.80, 2.47)	1.57 (0.90, 2.71)
∑DEHP^b^					
Q1	Ref	Ref	Ref	Ref	Ref
Q2	1.24 (0.79, 1.93)	1.63 (1.16, 2.28)	1.38 (0.96, 1.99)	1.12 (0.67, 1.88)	1.18 (0.79, 1.77)
Q3	1.23 (0.80, 1.90)	1.23 (0.81, 1.89)	0.86 (0.57, 1.31)	1.19 (0.69, 2.07)	1.53 (0.94, 2.49)
Q4	1.38 (0.90, 2.12)	1.45 (0.88, 2.41)	1.04 (0.66, 1.63)	1.24 (0.82, 1.88)	1.56 (0.93, 2.59)

^a^Adjusted for urinary creatinine, age, sex, race/ethnicity, total caloric intake, education, physical activity, smoking, and poverty

^b^Adjusted for covariates in a except sex

For women, higher urinary levels of MBzP were associated with an increased odds of central obesity (adj. POR for Q4 versus Q1: 1.71; 95 % CI: 1.07, 2.73) and hyperglycemia (adj. POR for Q2: 1.52; 95 % CI: 1.05, 2.18 and adj. POR for Q3: 2.06, 95 % CI: 1.31, 3.22 compared to Q1), with a suggestive association with hypertriglyceridemia (adj. POR for Q4 versus Q1: 1.75, 95 % CI: 0.98, 3.13). Although an association between urinary MBzP and MetS was not observed in men, exposure to the highest quartile of MBzP was associated with a significantly increased odds of central obesity (adj. POR: 1.71; 95 % CI: 1.06, 2.77), but not with other components of MetS. In men, urinary levels of ∑DEHP metabolites showed positive associations with central obesity (adj. POR for Q4 versus Q1: 1.68; 95 % CI: 1.02, 2.79) and high blood pressure (adj. POR for Q2: 1.59; 95 % CI: 1.05–2.40 and adj. POR for Q4 v. Q1: 1.85; 95 % CI: 1.12, 3.05). Suggestive findings were also present for higher ∑DEHP metabolite levels and hypertryglyceridemia in men (adj. POR for Q4 v. Q1: 1.55; 95 % CI: 0.97, 2.49). Weaker associations were found for ∑DEHP and these components in women, with only hypertriglyceridemia reaching statistical significance (adj. POR for Q2 v. Q1: 1.63; 95 % CI: 1.16, 2.28). When evaluating the number of components of MetS, only ∑DEHP was associated with a greater number of metabolic abnormalities in men, whereas among women, higher concentrations of MBzP, MnBP, MiBP, and ∑DEHP were associated with a greater number of MetS components (see Additional file 1: Table S2 for details).

In men, higher concentrations of MBzP were associated with an elevated odds of MetS only for younger men (adj. POR for Q4 of MBzP in men <50 years: 1.86; 95 % CI: 1.08, 2.55) (Table 5). A statistically significant inverse association was found for higher concentrations of MBzP relative to the lowest concentrations of this phthalate metabolite for older men. While only older men with concentrations in the low-moderate range for ∑DEHP showed a significant increased odds of MetS (adj. POR for men ≥50 years for Q2 versus Q1: 2.57; 95 % CI: 1.02, 1.69), even older men in the higher concentrations of ∑DEHP (Q3 and Q4) showed a suggested 2-fold increased odds of MetS compared to men in the lowest quartile of ∑DEHP. On the other hand, younger men with the highest concentrations of ∑DEHP had the strongest odds of MetS (adj. POR for men ≤50 years for Q4 versus Q1: 2.47; 95 % CI: 1.50, 3.62) (Table 5).

**Table 5:** Prevalence odds ratios and 95 % confidence intervals for metabolic syndromes stratified by age and sex

	Men		Women	
	<50 years	≥50 years	<50 years	≥50 years
	(*n* = 728)	(*n* = 660)	(*n* = 726)	(*n* = 605)
MBzP^a^				
Q1	Ref	Ref	Ref	Ref
Q2	1.75 (1.02, 2.43)	0.94 (0.41, 0.73)	1.45 (0.74, 2.83)	1.71 (1.00, 2.92)
Q3	1.53 (0.86, 198)	0.79 (0.38, 0.75)	2.25 (1.13, 4.49)	1.23 (0.57, 2.63)
Q4	1.86 (1.08, 2.55)	0.67 (0.35, 0.74)	1.84 (0.80, 4.22)	1.82 (0.86, 3.85)
∑DEHP^a^				
Q1	Ref	Ref	Ref	Ref
Q2	1.08 (0.57, 1.20)	2.57 (1.02, 1.69)	2.38 (1.37, 4.14)	1.14 (0.63, 2.07)
Q3	1.60 (0.76, 1.71)	2.00 (0.79, 1.31)	1.85 (1.02, 3.37)	1.23 (0.61, 2.50)
Q4	2.47 (1.50, 3.62)	2.07 (0.96, 1.80)	2.37 (1.13, 4.97)	1.00 (0.49, 2.06)

^a^ Prevalence odds ratios are adjusted for urinary creatinine, age, race/ethnicity, total caloric intake, education, physical activity, smoking, and poverty

In women, the association beween MBzP and MetS was somewhat similar when evaluating women who were <50 years versus ≥50 years of age (Table 5). On the other hand, the association between ∑DEHP and MetS was only seen among women <50 years. Specifically higher levels of ∑DEHP were associated with over a 2-fold increased odds of MetS compared to women who had the lowest levels of ∑DEHP (adj. POR for Q4: 2.37; 95 % CI: 1.13, 4.97). No associations reached statistical significance for women ≥50 years of age (*n* = 605).

**Fig. 1:**
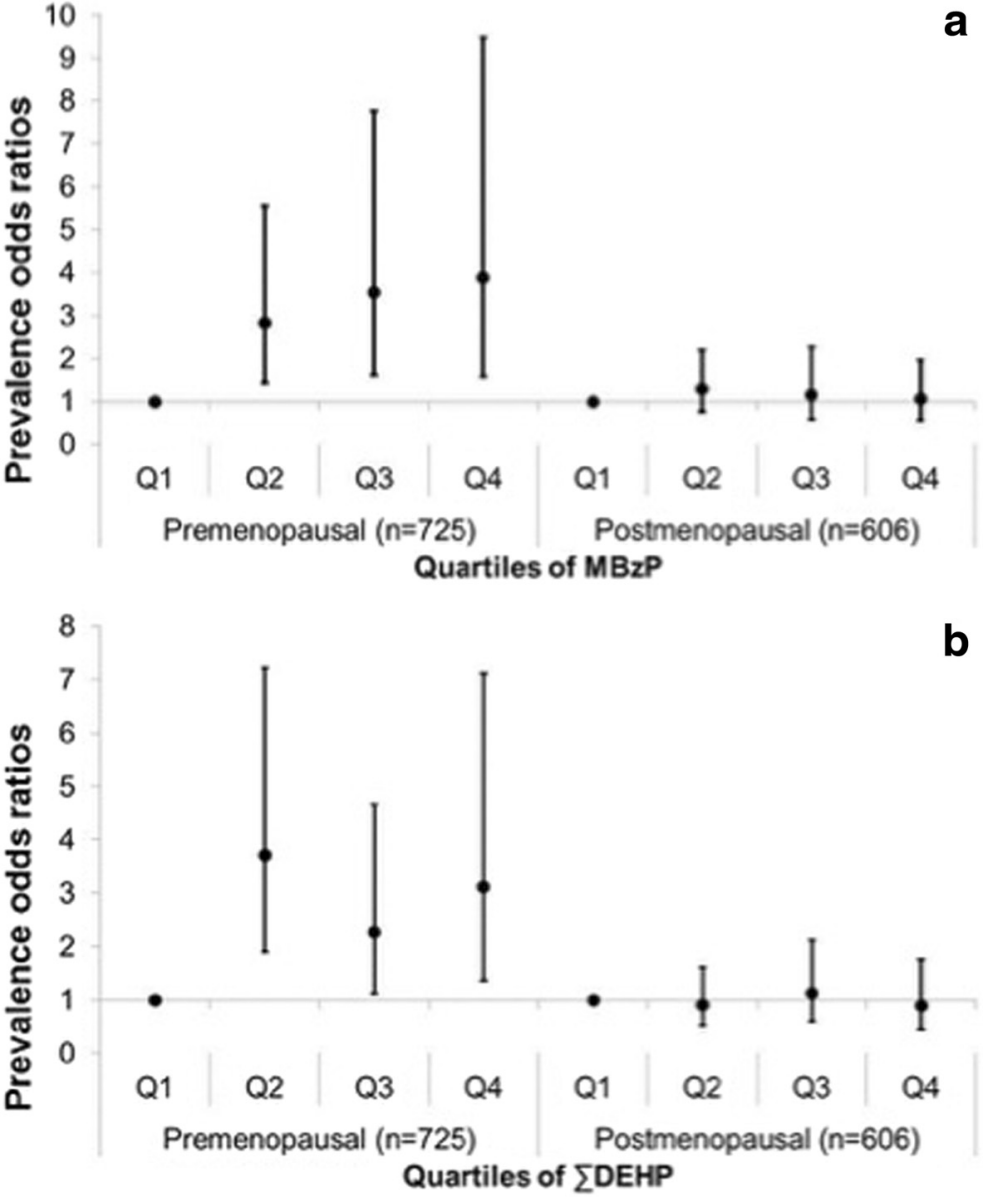
**a** Prevalence odds ratios for the association between urinary MBzP concentrations and metabolic syndrome stratified by menopausal status in women. **b** Prevalence odds ratios for the association between urinary ∑DEHP metabolites concentrations and metabolic syndrome stratified by menopausal status in women. Prevalence odds ratios were adjusted for urinary creatinine, age, race/ethnicity, total caloric intake, education, physical activity, smoking, and poverty

For premenopausal women (*n* = 725), strong positive associations were observed for MetS with both MBzP and ∑DEHP. In fact, associations for both of these phthalate metabolites were even stronger than that seen in age- and sex-stratified analyses. Specifically, pre-menopausal women with higher levels of MBzP had an ~3 to 4-fold increased odds of MetS compared to women with the lowest concentrations. Likewise, pre-menopausal women with higher concentrations of ∑DEHP had a 2 to 3-fold increased odds of MetS relative to women with the lowest concentrations of ∑DEHP. See Fig. 1. In contrast, no association was observed with these or other phthalate metabolites in postmenopausal women (*n* = 606) (Fig. 1).

## Discussion

In this study, we found higher levels of MBzP and ∑DEHP to be associated with a increased odds of MetS when compared to having the lowest concentrations in the overall study population. These associations held even after adjusting for a variety of sociodemographic and lifestyle factors. When stratifying by sex, higher levels of MBzP were associated with a greater odds of MetS in women, while higher levels of ∑DEHP were associated with an increased odds of MetS in men. The associations between higher levels of certain phthalates (i.e. MBzP and ∑DEHP) and MetS were stronger among men ≥50 years and women < 50 years. Despite having lower prevalence of MetS, the strongest association was seen among pre-menopausal women, with over 3-fold increased odds of MetS among those with the highest levels of MBzP or ∑DEHP compared to those with the lowest levels.

When assessing components of metabolic syndrome, previous studies have found associations between higher levels of certain phthalate metabolites and diabetes [2, 30, 31], insulin resistance [4, 6], obesity [1, 6, 40, 41], which are markers of cardiovascular risk [42–44]. For example, a previous study showed both MBzP and ∑DEHP to be associated with increased insulin resistance and fasting glucose levels among adult participants of NHANES 2001–2008 [4]. These associations differed in men and women, with a suggestion of a stronger association between MBzP and insulin resistance in women and a slightly stronger association between ∑DEHP and fasting glucose in men [4]. The present study also found similar associations of MBzP and ∑DEHP with hyperglycemia. The pattern of metabolites with sex was in similar directions as previously reported [4]. As such, this growing body of evidence suggests that butylbenzyl phthalate, the parent compound of MBzP, and DEHP may increase the odds of MetS through mechanisms related to glucose dysregulation. Since the bulk of the studies are cross-sectional, like the present study, it is unclear whether higher butylbenzyl phthalate and DEHP levels precede insulin resistance or hyperglycemia or a consequence of these conditions, possibly attributed to differences in phthalate sources, such as increased medication use. As such, other pathways cannot be ruled out, nor can reverse causation. Future prospective studies will need to evaluate the exact mechanism by which these parent compounds might operate to increase the risk of MetS.

A number of NHANES studies have documented an association between higher phthalate metabolite concentrations and increased obesity and waist circumference [6, 40, 41]. However, these findings differ by sex, with higher concentrations of certain phthalate metabolites being associated with obesity and waist circumference only in men [1]. The present study found similar associations in both men and women for MBzP with a ~70 % increased odds of central obesity based on MetS criteria for both men and women. In fact, this component of MetS was most strongly associated with higher phthalate metabolite concentrations. The hypothesized pathway by which phthalates are thought to alter adiposity is through their ability to bind to PPAR-γ and upregulate target genes associated with adipogenesis in an environment of excess caloric intake [29]. It is also plausible that central obesity was strongly associated with MBzP and DEHP due to greater intake of high fat foods that may have been packaged in phthalate-containing plastics, which would contribute to greater fat mass. Prospective studies, evaluating potential mechanisms, are needed to disentangle these associations.

Higher levels of ∑DEHP were associated with hyperglycemia and hypertriglyceridemia in the overall study population. ∑DEHP was also associated with increased central obesity and hypertension in men. A study of elderly Swedish men and women found an association between ∑DEHP and elevated LDL and carotid artery plaques, a sign of cardiovascular disease [42]. Our findings suggest that other CV risk factors, namely decreased HDL cholesterol and hypertension, might be associated with higher phthalate exposure. Future work will need to explore whether phthalates might be associated with cardiovascular disease, a leading cause of mortality in the U.S.

In the present study, analyses were stratified by several factors, including sex, age, and menopausal status, as phthalate metabolite concentrations and the prevalence of MetS may differ by these [4, 25, 33, 34]. Associations were stronger among young women and older men. It is possible that these differences are due to differing hormonal states and possibly by the effects of aging. In women, we saw that independent of age, there was a different association between certain phthalate metabolites and MetS based on menopausal status. However, the reasons for sex and age differences are unclear and require further investigation. Based on data from NHANES, U.S. women have higher concentrations of MEP, MBzP, MnBP, MiBP, MCPP, and ∑DEHP compared to U.S. men [25]. From the same study, urinary phthalate metabolite concentrations were lower in those ≥40 years of age than in those <20 years of age [25]. Based on another previous study using NHANES, higher concentrations of DEHP metabolites were associated with earlier age at menopause among women who participated in NHANES [45]. While it is possible that phthalates may modulate estrogen levels [46], phthalates could also affect age at menopause, with implications for conditions that are more prevalent in post-menopausal women. Future studies will need to elucidate these associations to better understand these potential differences by sex, age, and menopausal status.

Butyl benzyl phthalate, the parent compound of MBzP is commonly found in personal care products, food packaging, and industrial solvents [24]. While ∑DEHP was once thought to be of greatest concern due to having many common sources of exposure, such as polyvinyl plastics containing this phthalate parent compound, more recently other metabolites, such as MBzP have been seen as an important phthalate metabolite [24]. Although there have been some decreases in exposure to certain phthalates over time [47], these chemicals are still used in consumer products and are being replaced by similar chemicals without known health effects. In fact, a recent study found similar associations with a replacement compound, diisononyl phthalate [7].

This study has several limitations. First, due to the cross-sectional study design, we are unable to establish temporality and could not rule out the possibility of reverse causation. For example, higher phthalate metabolite concentrations could be a consequence of having MetS rather than higher phthalate metabolite concentrations influencing MetS. Second, this study used a one-time measurement of phthalate metabolite levels in the study population. A single fasting measure may not adequately capture overall exposure to urinary phthalate metabolites, leading to non-differential misclassification. As such, we may underestimate the associations between urinary phthalate metabolites and metabolic syndrome in this study population. Phthalates are non-persistent chemicals and are rapidly excreted within 24–48 h [24]. These chemicals are known to vary within a person over time and have a moderate reliability over time with intraclass correlations for creatinine-adjusted urinary phthalate metabolite concentrations ranging from 0.39 to 0.55 based on women participating in the Nurses’ Health Study [48]. Another study found very low reproducibility with intraclass correlations ranging from 0.03 to 0.20; however, these study participants were all living in Shanghai, which may have different patterns of exposure compared to individuals living in the U.S. [49]. Given that MetS may take years or decades to develop, the short half-life of these chemicals may only be relevant, if exposure to higher concentrations of phthalates is on average consistent across time. Future studies will need to evaluate repeated measures of these chemicals across multiple time points to determine their association with MetS and its components.

Third, due to the cross-sectional nature of the study, we were unable to determine whether higher exposure to these chemicals was attributed to exposures that were the effect of MetS, such as increased medication and personal care product use, as well as intake of food products packaged in DEHP-containing plastics or foods that are high in fat, such as protein, dairy, and cooking oil [24]. However, adjusting for the sources of exposure would not yield an accurate measure of association Fourth, the fact that study participants fasted could underestimate the associations for ∑DEHP and MetS, as concentrations may be lower due to not having taken foods that are a primary source of exposure to this parent compound. Finally, the lack of dose–response associations could either signal threshold effects, the possibility of negative feedback loops, or residual confounding.

Despite these limitations, the present study has several strengths. First, this study was able to evaluate 5 different phthalate metabolites, as well as a summary measure of the DEHP metabolites and their associations with MetS among a substantial study population of U.S. adults. Second, data were utilized from a representative sample of the U.S. population that was comprised of both men and women that was racially/ethnically diverse. Third, analyses were stratified by sex and age, as well as menopausal status, to determine whether associations differed by subgroups based on a priori hypotheses. Fourth, the dataset utilized the widely accepted criteria for MetS based on the NCEP/ATPIII classification. These findings provide insights into a prevalent chemical exposure on MetS as a cardiovascular risk marker.

## Conclusion

In conclusion, in this cross-sectional study, we found MBzP and ∑DEHP to be associated with an increased odds of MetS. The associations suggestively differed between men and women depending on the phthalate metabolite studied and by each component of MetS. Associations appeared to be the strongest for premenopausal women with higher levels of MBzP and ∑DEHP and MetS. These findings suggest further evaluating the role of phthalate metabolite concentrations prospectively to determine whether these chemicals can alter the risk of MetS among individuals without the condition. Furthermore, additional work is needed to understand the roles of sex, age, and menopausal status on the associations between phthalate metabolite levels and MetS. If replicated, these findings suggest that phthalates could be a potentially modifiable risk factor of MetS.

## Additional file

Additional file 1:
**Table S1.** Prevalence odds ratios and 95 % confidence intervals for individual components of metabolic syndrome according to urinary phthalate metabolite levels. **Table S2.** Prevalence odds ratios and 95 % confidence intervals for the number of metabolic syndrome components according to urinary phthalate metabolites in multivariable ordinal logistic regression. (DOC 79 kb)

## Abbreviations

CI: confidence interval
DEHP: di-(2-ethylhexyl) phthalate
LOD: limit of detection
MBzP: mono-benzyl phthalate
MCPP: mono-(3-carboxypropyl) phthalate
MEHHP: mono-(2-ethyl-5-hydroxyhexyl) phthalate
MEHP: mono-(2-ethylhexyl) phthalate
MEOHP: mono-(2-ethyl-5-oxohexyl) phthalate
MEP: mono-ethyl phthalate
MetS: metabolic syndrome
MiBP: mono-isobutyl phthalate
MnBP: mono-n-butyl phthalate
NHANES: National Health and Nutrition Examination Survey
POR: prevalence odds ratio
Q: quartile
