# Supplementary material for: The association between phthalates and metabolic syndrome: the National Health and Nutrition Examination Survey 2001–2010
Source: Environ Health. 2016 Apr 14;15:52. doi: 10.1186/s12940-016-0136-x (PMC4832560; doi:10.1186/s12940-016-0136-x)
Supplement: Additional file 1: — Table S1. Prevalence odds ratios and 95 % confidence intervals for individual components of metabolic syndrome according to urinary phthalate metabolite levels. Table S2. Prevalence odds ratios and 95 % confidence intervals for the number of metabolic syndrome components according to urinary phthalate metabolites in multivariable ordinal logistic regression. (DOC 79 kb) [file 12940_2016_136_MOESM1_ESM.doc]

| **Table S1. Prevalence odds ratios and 95% confidence intervals for individual components of metabolic syndrome according to urinary phthalate metabolite levels** | | | | | |
| --- | --- | --- | --- | --- | --- |
|  | Individual components of metabolic syndrome | | | | |
|  | Central obesity | Hypertriglyceridemia | Low HDL cholesterol | High blood pressure | Hyperglycemia |
|  | Overall population | | | | |
| Cases/total n | 1522/2719 | 849/2719 | 816/2719 | 871/2719 | 1289/2719 |
| MEP^1^ |  |  |  |  |  |
| Q1 | Ref | Ref | Ref | Ref | Ref |
| Q2 | 1.11 (0.81, 1.52) | 0.93 (0.68, 1.26) | 0.87 (0.64, 1.19) | 1.07 (0.78, 1.48) | 1.25 (0.95, 1.64) |
| Q3 | 1.05 (0.79, 1.39) | 0.93 (0.70, 1.25) | 0.83 (0.61, 1.14) | 0.93 (0.72, 1.20) | 0.99 (0.73, 1.33) |
| Q4 | 1.04 (0.79, 1.37) | 0.97 (0.72, 1.31) | 0.69 (0.51, 0.95) | 1.39 (0.99, 1.96) | 1.01 (0.75, 1.37) |
| MnBP^1^ |  |  |  |  |  |
| Q1 | Ref | Ref | Ref | Ref | Ref |
| Q2 | 1.30 (1.01, 1.66) | 1.23 (0.93, 1.62) | 1.05 (0.79, 1.40) | 1.03 (0.75, 1.42) | 1.01 (0.76, 1.33) |
| Q3 | 1.24 (0.97, 1.57) | 0.94 (0.70, 1.28) | 0.76 (0.53, 1.08) | 1.09 (0.72, 1.64) | 1.25 (0.96, 1.64) |
| Q4 | 1.37 (0.96, 1.96) | 1.23 (0.86, 1.75) | 0.93 (0.63, 1.36) | 1.09 (0.73, 1.63) | 1.02 (0.73, 1.45) |
| MCPP^1^ |  |  |  |  |  |
| Q1 | Ref | Ref | Ref | Ref | Ref |
| Q2 | 1.15 (0.83, 1.60) | 1.24 (0.93, 1.67) | 1.12 (0.79, 1.58) | 1.06 (0.75, 1.50) | 1.00 (0.75, 1.34) |
| Q3 | 1.17 (0.83, 1.65) | 1.30 (0.95, 1.79) | 1.30 (0.90, 1.89) | 0.91 (0.63, 1.32) | 1.04 (0.74, 1.46) |
| Q4 | 1.28 (0.96, 1.71) | 1.09 (0.77, 1.53) | 1.26 (0.87, 1.81) | 0.95 (0.68, 1.34) | 1.44 (1.02, 2.03) |
| MiBP^1^ |  |  |  |  |  |
| Q1 | Ref | Ref | Ref | Ref | Ref |
| Q2 | 1.30 (1.01, 1.69) | 1.01 (0.74, 1.37) | 0.94 (0.70, 1.26) | 1.17 (0.86, 1.60) | 1.25 (0.86, 1.82) |
| Q3 | 1.40 (1.06, 1.86) | 0.71 (0.54, 0.94) | 0.81 (0.60, 1.10) | 1.06 (0.68, 1.66) | 1.38 (0.95, 2.00) |
| Q4 | 1.62 (1.22, 2.15) | 0.66 (0.47, 0.92) | 0.94 (0.69, 1.29) | 1.34 (0.92, 1.96) | 2.46 (1.61, 3.74) |
|  |  |  |  |  |  |
| ^1^Adjusted for urinary creatinine, age, sex, race/ethnicity, total caloric intake, education, physical activity, smoking, and poverty | | | | | |

| **Table S2. Prevalence odds ratios and 95% confidence intervals for the number of metabolic syndrome components according to urinary phthalate metabolites in multivariable ordinal logistic regression** | | | |
| --- | --- | --- | --- |
|
|  | Overall population | Men | Women |
|  | (n=2719) | (n=1388) | (n=1331) |
|  | Multivariable OR (95% CI) | | |
| MEP |  |  |  |
| Q1 | Ref | Ref | Ref |
| Q2 | 1.09 (0.85, 1.39) | 0.97 (0.68, 1.37) | 1.29 (0.92, 1.80) |
| Q3 | 0.92 (0.71, 1.19) | 0.89 (0.61, 1.31) | 1.00 (0.69, 1.45) |
| Q4 | 1.01 (0.79, 1.28) | 1.02 (0.68, 1.53) | 1.13 (0.78, 1.63) |
| MBzP |  |  |  |
| Q1 | Ref | Ref | Ref |
| Q2 | 1.15 (0.91, 1.46) | 1.08 (0.73, 1.58) | 1.13 (0.82, 1.55) |
| Q3 | 1.30 (0.98, 1.72) | 1.00 (0.67, 1.49) | 1.55 (1.06, 2.28) |
| Q4 | 1.58 (1.16, 2.13) | 1.20 (0.85, 1.71) | 1.85 (1.16, 2.95) |
| MnBP |  |  |  |
| Q1 | Ref | Ref | Ref |
| Q2 | 1.14 (0.94, 1.40) | 1.05 (0.79, 1.40) | 1.16 (0.87, 1.55) |
| Q3 | 1.08 (0.86, 1.35) | 0.81 (0.58, 1.14) | 1.38 (1.01, 1.89) |
| Q4 | 1.14 (0.84, 1.55) | 1.38 (0.89, 2.14) | 0.95 (0.62, 1.47) |
| MCPP |  |  |  |
| Q1 | Ref | Ref | Ref |
| Q2 | 1.17 (0.90, 1.53) | 1.07 (0.74, 1.54) | 1.19 (0.82, 1.71) |
| Q3 | 1.24 (0.93, 1.66) | 1.01 (0.66, 1.56) | 1.28 (0.84, 1.96) |
| Q4 | 1.33 (1.03, 1.73) | 1.22 (0.85, 1.75) | 1.28 (0.85, 1.91) |
| MiBP |  |  |  |
| Q1 | Ref | Ref | Ref |
| Q2 | 1.20 (0.93, 1.55) | 0.96 (0.71, 1.30) | 1.35 (0.95, 1.93) |
| Q3 | 1.06 (0.81, 1.39) | 0.88 (0.61, 1.27) | 1.22 (0.84, 1.78) |
| Q4 | 1.41 (1.08, 1.84) | 1.13 (0.80, 1.59) | 1.53 (1.04, 2.26) |
| DEHP |  |  |  |
| Q1 | Ref | Ref | Ref |
| Q2 | 1.55 (1.23, 1.95) | 1.42 (1.03, 1.94) | 1.48 (1.11, 1.98) |
| Q3 | 1.46 (1.14, 1.86) | 1.47 (1.05, 2.05) | 1.27 (0.89, 1.83) |
| Q4 | 1.77 (1.37, 2.30) | 1.80 (1.25, 2.60) | 1.51 (1.02, 2.22) |
| ^1^Adjusted for urinary creatinine | | |  |
| ^2^Adjusted for urinary creatinine, age, sex, race/ethnicity, total caloric intake, education, physical activity, smoking, and poverty | | | |
| ^3^Adjusted for the same variables in (2) except sex | | |  |
